# The clinical significance of accompanying NME on preoperative MR imaging in breast cancer patients

**DOI:** 10.1371/journal.pone.0178445

**Published:** 2017-05-30

**Authors:** Hye Mi Gweon, Joon Jeong, Eun Ju Son, Ji Hyun Youk, Jeong-Ah Kim, Kyung Hee Ko

**Affiliations:** 1 Department of Radiology, Gangnam Severance Hospital, Yonsei University College of Medicine, Seoul, Republic of Korea; 2 Department of Surgery, Gangnam Severance Hospital, Yonsei University College of Medicine, Seoul, Republic of Korea; 3 Department of Radiology, CHA Bundang Medical Center, CHA University, School of Medicine, Gyeonggi-do, Republic of Korea; University of South Alabama Mitchell Cancer Institute, UNITED STATES

## Abstract

**Purpose:**

To investigate the significance of accompanying NME in invasive ductal carcinoma (IDC) on preoperative MR imaging and assess the factors affecting the significance.

**Methods:**

Between January 2015 and February 2016, 163 consecutive patients with IDC who underwent preoperative MR imaging and subsequent surgery were enrolled and reviewed. Index cancer mass size and total extent with accompanying NME on MR images was measured and compared with pathologic size. Positive NME was defined as pathological result of IDC or DCIS. To identify affecting factors associated with frequency of accompanying NME on MR and positive pathologic result, clinicopathologic features were compared between breast cancers with NME and without NME, and between breast cancers with positive NME and negative NME using the Student t-test or Chi-square test.

**Results:**

Of the 163 invasive breast cancers, 123(75.5%) cancers presented as only mass feature and 40(24.5%) cancers had accompanying NME around the index mass. Of the 40 accompanying NME, 22 (55%) had positive pathologic results and 18 (45%) had negative results. The HER2 positive status was significantly associated with positive pathologic results of accompanying NME (*P* = .016).

**Conclusion:**

Accompanying NME on preoperative MR imaging showed malignant pathologic results in 55%. The HER2 positive IDC was more frequently accompanied by malignant NME.

## Introduction

Preoperative breast magnetic resonance (MR) imaging has been routinely used for extent of disease assessment in patients with newly diagnosed breast cancer. MR imaging is considered the most accurate of the imaging modalities for breast cancer evaluation, capable of identifying multifocal/multicentric or contralateral breast malignancies not evident by conventional imaging [1–5]. Moreover, MR imaging offers more accurate local extent of invasive breast cancer and in situ tumors than ultrasound and mammography [6,7].

However, controversy still exists about the proper use of preoperative MR examination in newly diagnosed breast cancer. MR imaging has a low specificity leading to more imaging, biopsies, and more aggressive surgery. [1,2,8]. In addition, overestimation of the cancer extent can cause wider excision and conversion to mastectomies [1,2,9]. The exact reasons for overestimation of cancer extent are not fully understood. Grimsby et al. reported MR imaging overestimated 33% of tumors. Among them, 65% had additional significant findings in the breast tissue around the main lesion: satellite lesions, ductal carcinoma in situ (DCIS), and lymphovascular invasion [10]. Recent study also reported that DCIS histology was strongly associated with discordance between MR imaging and pathology size of breast cancer [11]. The most common morphologic features of DCIS are nonmass enhancement (NME) on MR images [12–15]. Although segmental, clumped, and linear NME is associated with malignancy, NME is causing a high proportion of false-positive diagnoses on breast MR imaging [16]. The invasive ductal carcinoma (IDC) most commonly presents as an enhancing mass on MR images, it is occasionally associated with NME surrounding the index breast cancer mass. However, to the best of our knowledge, there has been no data regarding the significance of accompanying NME on preoperative MR imaging. Determining impact of NME is critical to ensure proper surgical planning in breast cancer. Therefore, the purpose of this study was to investigate the significance of accompanying NME in invasive ductal breast cancer on preoperative MR imaging and assess the factors affecting the significance.

## Materials and methods

### Subject population

The institutional review board of our institution (Gangnam Severance Hospital) approved this retrospective analysis, and the need for informed consent was waived. Between January 2015 and February 2016, 204 consecutive patients with newly diagnosed invasive ductal carcinoma (IDC) who underwent preoperative MR imaging and subsequent surgery were reviewed. Among them, 31 patients were excluded due to neoadjuvant chemotherapy (n = 22) or post-excisional MR examination (n = 9). Of those, our study included breast cancer presented as mainly mass feature and with or without accompanying NME on preoperative MR imaging. Therefore, 10 patients with mainly NME feature breast cancer were excluded. Finally, 163 patients (median age, 54 years; range, 32 to 79 years) comprised our study population.

### MR imaging evaluation

All MR examinations were performed using a 3.0-T MR imager (Achieva; Philips Medical System, Best, Netherlands) with a dedicated, sensitivity encoding (SENSE)–enabled, four-channel breast coil in the prone position. All images were acquired with bilateral axial views. The routine protocol included turbo spin-echo T1- and T2-weighted sequences and a T2-weighted fat suppressed spin echo series. Dynamic contrast- enhanced MR examination included one pre-contrast and five post-contrast series using a fat-suppressed T1-weighted gradient echo sequence (TR/TE: 4.9/2.4; matrix, 340x340; flip angle, 12°; field of view, 34x34 cm; sliced thickness, 1.5 mm). Acquisition time of each post-contrast series was 74 seconds. Gadobutrol (Gadovist, Bayer Healthcare, Berlin, Germany) with a dose of 0.1 mmol/kg was injected using an automated injector (Nemoto; Nemoto Kyorindo, Tokyo, Japan) at a rate of 2 mL/sec, followed by a 20-mL saline flush.

MR imaging was retrospectively interpreted by two radiologists in consensus according to the BI-RADS^®^ Atlas without information of histopathology. Maximum diameter measurements were assessed by using a combination of precontrast and early post-contrast fat-suppressed T1-weighted and subtraction images. First, only size of index cancer mass was measured in the largest dimension among transverse, sagittal and coronal planes. When the lesion consisted of multiple mass lesions, the maximal diameter was not the sum of their diameter, but a single largest diameter was measured. Second, total extent of mass and accompanying NME was measured. Accompanying NME was defined NME lesions around the index mass less than 1cm apart. Kinetic feature was assessed by drawing a region of interest over the most suspicious portion of the lesion to measure the signal intensity change through dynamic images on Picture Archiving and Communication System (PACS).

### Pathologic assessment

Histologic evaluation was performed by one pathologist with 30 years’ experience in breast pathology. Surgical specimens were sliced into 5 mm thick sections that were fixed in formalin, embedded in paraffin, and stained with hematoxylin and eosin for microscopic evaluation. Tumor size was measured at the level of the largest diameter. The two histopathological tumor sizes were noted, 1) diameter of invasive tumor and 2) diameter of invasive and in situ carcinoma. Grading for invasive carcinoma was performed according to Elaston and Ellis [17] and for DCIS according to the grading part of the Van Nuys Classification [18]. The expression of estrogen receptor (ER), progesterone receptor (PR), and human epidermal growth factor 2 (HER2) was evaluated by the standard avidin-biotin complex immunohistochemical staining method. ER and PR was determined by nuclear staining, which was graded from 0 to 8 using the Allred score [19]. The results were categorized as positive when the total score, expressed as the sum of the proportion score and intensity score, was 3 or greater. The intensity of c-erbB-2 staining was scored as 0, 1+, 2+, or 3+. Tumors with a 3+ score were classified as HER2 positive, and tumors with a 0 or 1+ score were classified as negative. In tumors with a 2+ score, gene amplification using fluorescence in situ hybridization was used to determine HER2 status. The ratios of HER2 gene copies to the centromeric region of chromosome 17 ratios of 2.0 or more were interpreted as amplified [20].

### Data and statistical analysis

Preoperative tumor size on MR imaging was compared to pathologic tumor size. Index mass size on MR was compared to invasive carcinoma size on pathology. Total extent of mass and accompanying NME on MR was compared to IDC and DCIS size on pathology. Tumor size measurement on MR imaging within 5 mm of histopathological measurement were considered concordant.

After comparing the size between by MR and pathology, accompanying NME results was assessed. Positive NME was defined as pathological result of IDC or DCIS. To identify affecting factors associated with frequency of accompanying NME on MR and positive pathologic result, clinicopathologic features were compared between breast cancers with NME and without NME, and between breast cancers with positive pathologic result and negative pathologic result of NME using the Student t-test or Chi-square test.

All statistical analyses were performed using statistical software (SPSS, version 20.0; SPSS, Chicago, IL, USA). A *P* value of less than 0.05 was considered to indicate a significant difference. Confidence intervals (CIs) are shown at the 95% confidence level.

## Results

Of the 163 invasive breast cancers, 123(75.5%) cancers presented as only mass feature and 40(24.5%) cancers had accompanying NME around the index mass (Table 1). Index mass size was 2.0±1.0 cm in breast cancers without NME and 2.2 ± 0.9 cm in caners with accompanying NME on MR images. The concordance rate within 5 mm between mass size by MR and IDC size by pathology was 91.9% (113 of 123) in breast cancer without NME and 72.5% (29 of 40) in cancer with accompanying NME, there was significant difference (*P* = .005). The enhancement pattern and HER2 status were significantly difference between breast cancer with NME and without NME.

**Table 1:** Characteristics of patients without NME and with accompanying NME in MR imaging.

	Without NME (n = 123)	With NME (n = 40)	P-value
Age (y)	53.9 ±11.1	54.3 ± 9.0	.848
MR finding			
Index mass size (cm)	2.0 ± 1.0	2.2 ± 0.9	.135
Background parenchymal enhancement			.126
Minimal	57 (46.3)	24 (60)	
Mild	38 (30.9)	9 (22.5)	
Moderate	17 (13.8)	7 (17.5)	
Marked	11 (8.9)	0 (0)	
Shape of index mass			.438
Oval	23 (18.7)	4 (10)	
Round	11 (8.9)	4 (10)	
Irregular	89 (72.4)	32 (80)	
Margin of index mass			.248
Circumscribed	13 (10.6)	1 (2.5)	
Irregular	74 (60.2)	28 (70)	
Spiculated	36 (29.3)	11 (27.5)	
Enhancement of index mass			.017
Homogeneous	25 (20.5)	2 (5)	
Heterogeneous	87 (71.3)	30 (75)	
Rim	10 (8.2)	8 (20)	
Histopathologic result			
Invasive carcinoma size (cm)	1.9 ± 1.0	2.2 ± 1.2	.054
MR-pathology discrepancy			.005
Concordance	113 (91.9)	29 (72.5)	
Discordance	10 (10)	11 (27.5)	
Estrogen receptor status			.214
Positive	94 (76.4)	26 (65)	
Negative	29 (23.6)	14 (35)	
Progesterone receptor status			.150
Positive	72 (58.5)	19 (47.5)	
Negative	51 (41.5)	21(52.5)	
HER2 receptor status			.007
Positive	16 (13)	13 (32.5)	
Negative	107 (87)	27 (67.5)	

Table 2 lists the clinicopathologic findings of 40 breast cancers with accompanying NME on preoperative MR imaging. The total extent with NME on MR images was 4.5 ± 1.1 cm and pathologic size of IDC with associated DCIS was 3.2 ± 1.4 cm. There was significant difference between size by MR and by pathology (*P* < .001). Of the 40acccompanying NME, 22 (55%) had positive pathologic results and 18 (45%) had negative results. Among 22 NME with positive pathologic results, 4 were invasive carcinoma, 8 were high grade DCIS and 10 were intermediate grade DCIS. There was no significant difference in age and characteristics of MR findings between positive and negative results of NME. The HER2 receptor status was significantly associated with pathologic results of NME. Accompanying NME in HER2 positive breast cancer was more frequently had positive pathologic results than HER2 negative breast cancers (84.6% [11 of 13] *vs*. 40.7% [11 of 27], *P* = .016).

**Table 2:** Characteristics of patients with accompanying NME according to pathologic results.

	Positive (n = 22)	Negative (n = 18)	P-value
Age (y)	53.0±8.1	55.8±10.0	.262
Age category			
<50 years	7 (31.8)	6 (33.3)	.999
≥ 50 years	15 (68.2)	12 (66.7)	
MR finding			
Index mass size (cm)	2.3 ± 1.0	2.2 ± 0.7	.748
Total extent with NME (cm)	4.7 ± 1.3	4.3 ± 0.8	.351
Background parenchymal enhancement			
Minimal	12 (54.5)	12 (66.7)	.687
Mild	6 (27.3)	3 (16.7)	
Moderate	4 (18.2)	3 (16.7)	
Distribution of NME			
Focal	5 (22.7)	3 (16.7)	.499
Linear	4 (18.2)	5 (27.8)	
Segmental	11 (50.0)	6 (33.3)	
Regional	2 (9.1)	4 (22.2)	
Enhancement of NME			
Homogeneous	1 (4.5)	4 (22.2)	.056
Heterogeneous	17 (77.3)	14 (77.8)	
Clumped	4 (18.2)	0 (0)	
Histopathologic result			
Invasive carcinoma size (cm)	2.5 ± 1.4	2.0 ± 0.7	.158
Estrogen receptor status			
Positive	15 (68.2)	11 (61.1)	.744
Negative	7 (31.8)	7 (38.9)	
Progesterone receptor status			.525
Positive	9 (40.9)	10 (55.6)	
Negative	13 (59.1)	8 (44.4)	
HER2 receptor status			.016
Positive	11 (50)	2 (11.1)	
Negative	11 (50)	16 (88.9)	

## Discussion

In our study, we found that 24.5% IDC with mass feature was accompanied by NME on preoperative MR imaging. Among them, 55% accompanying NME had malignant pathologic results. Especially, HER2 positivity was significantly associated with malignant pathologic results of NME. The concordance rate within 5 mm between mass size by MR and invasive size by pathology was 91.9% in IDC without NME and 72.5% in IDC with accompanying NME, these rates were relatively high compared with previous studies. There are several studies about the accuracy of breast MR in estimating tumor extent. Onesti et al. found that MR imaging significantly overestimated mean tumor size and overall concordance rate was 57.1% [21]. Grimsby et al. also found that 53% of concordance rate between MR and pathologic cancer size within 0.5 cm and 33% was overestimated [10]. However, their study did not consider the morphologic feature on MR images. A recent study reported that NME significantly predicted the discordance between MR image and pathology for sizing of breast cancer [22]. They found that mass lesions were overestimated in 7% and NME were in 41%. In our study, we compared size of index mass on MR with invasive tumor size on pathology. This point was maybe cause the high concordant rate.

Tumor size is one of the most important factors for making assessment and surgical management of breast cancer. Especially, accurate preoperative assessment of exact cancer extent is crucial for deciding breast conserving surgery. The positive resection margin is associated with a local recurrence and reoperation [23,24]. A recent large cohort study reported that DCIS was associated with positive resection margin [25]. Another study also found that DCIS is the strongest independent predictor of discrepancy between MR image and pathology sizing of breast cancer [11]. DCIS lesions have been found to exhibit NME at a high rate on MR images [12,26]. NME were the known major cause of false-positive breast MR findings. A study reported that the false positive rate of NME was 48%, it is significantly high rate compared with mass lesions [16]. In other words, their study reported that the positive predictive value for NME lesions were 51.7%. This value is comparable with our results (55%) and, it is suitable for BI-RADS category 4 that shouldn’t be ignored finding in MR imaging.

Drawbacks to the high false positive rate of NME on MR images, it is necessary to find the affecting factors associated with increase positive predictive values. In our study, positive HER2 status was significantly associated with positive pathologic result of NME. The characteristics of NME including distribution and enhancement pattern were not significantly associated with pathologic results. There was no previous study about the association with hormonal subtype and significance of NME. Further study about affecting factors the significance of NME and cancer extent measurement including hormonal subtype is necessary.

Our study only included IDC, not including ILC. Because, ILC frequently manifests as focal or regional NME on preoperative MR images, it is different from IDC [27]. Therefore, distinct study should be investigated according the type of breast cancer for more individual accurate preoperative assessment.

This study has several limitations. First, this was a retrospective study from a single institution with a relatively small number of patients. The final result was investigated from the comparison between MR images and pathologic report without direct preoperative mapping. Therefore, in case of negative results, we did not know what kind of benign pathology was. Second, clinical effects of accompanying NME including change of surgical plan, the rate of positive resection margin and recurrence rate were not evaluated.

Despite these limitations, our data demonstrated that accompanying NME with IDC on preoperative MR images had clinically significant result, yielding a 55% positive pathologic results. The HER 2 positive IDC was more frequently associated with malignant NME. Our result suggests that the accompanying NME should be carefully investigated on preoperative MR images and individually determined according to molecular subtypes. These observations may inform future clinical practice validated in prospective trials.

## Supporting information

S1 File Patient’s data of clinicopathologic and preoperative MR findings. (XLSX)
